# Contrasting roles of secondary motor cortex projections to the dorsolateral and dorsomedial striatum in incubation of cocaine-seeking

**DOI:** 10.1038/s41386-026-02385-3

**Published:** 2026-03-25

**Authors:** Víctor Gómez-Pineda, Donald Huang, Yingying Chen, Changyong Guo, Yao-Ying Ma

**Affiliations:** 1 Department of Biochemistry, Molecular Biology and Pharmacology, Indiana University School of Medicine, Indianapolis, IN USA; 2 Stark Neurosciences Research Institute, Indiana University School of Medicine, Indianapolis, IN USA

**Keywords:** Synaptic transmission, Neurophysiology

## Abstract

Cocaine use disorder is characterized by persistent relapse vulnerability, which escalates with prolonged withdrawal. Corticostriatal circuits are key substrates for relapse, yet the role of the secondary motor cortex (M2) and its distinct projections to the dorsolateral striatum (DLS) and dorsomedial striatum (DMS) remains poorly understood. Here, we combined intravenous self-administration, pathway-selective optogenetic inhibition, and ex vivo patch-clamp recordings to measure comprehensive synaptic responses during withdrawal. Relative to withdrawal day 1, rats exhibited cocaine seeking incubation on withdrawal day 45, which was therefore selected as the primary experimental time point. Whole-cell recordings revealed heightened intrinsic excitability of M2 cortical pyramidal neurons and increased frequency, but not amplitude, of spontaneous excitatory postsynaptic currents (sEPSCs) in both DLS and DMS medium-sized spiny neurons (MSNs), suggesting the possibility of enhanced presynaptic glutamate release. Optogenetic inhibition of eNpHR-expressing M2 terminals produced opposite behavioral outcomes: suppression of cocaine seeking when targeted to the DLS, but paradoxical enhancement when targeted to the DMS. In cocaine-exposed, but not saline, rats, optogenetic inhibition increased sEPSC frequency in both DLS and DMS MSNs, suggesting altered integration of M2 or local inhibitory inputs and non-M2 excitatory afferents. Inhibitory adaptations diverged across striatal subregions: in the DLS, repeated inhibition persistently increased spontaneous inhibitory postsynaptic current (sIPSC) frequency, whereas in the DMS, sIPSC enhancement was transient, with frequency and amplitude increasing only during the first light-on period, but amplitude rapidly declined thereafter. Together, these findings suggest that M2-striatal projections contribute in a pathway-specific manner to relapse vulnerability and are associated with alterations in excitatory-inhibitory balance within M2-DLS and M2-DMS circuits.

## Introduction

Cocaine use disorder is characterized by persistent vulnerability to relapse even after extended periods of abstinence. Preclinical studies have demonstrated that relapse risk increases with the length of withdrawal, a phenomenon termed the incubation of cocaine craving [1]. This behavioral time course suggests that enduring neuroadaptations emerge during abstinence, particularly within corticostriatal circuits that govern drug seeking. Identifying the circuit- and synapse-specific regulation underlying this incubation process is critical for developing strategies to prevent relapse.

The secondary motor cortex (M2) is functionally well positioned to contribute to addiction-related behaviors. Drug seeking and taking are often characterized by extremely short latencies after exposure to drug-associated cues, reflecting rapid, automatic actions that bypass intentional or volitional control [2]. Repeated drug exposure also produces locomotor sensitization [3, 4], a well-studied motor adaptation that further implicates motor circuits in addiction. Although locomotor activity itself is only weakly coupled to craving [5], drug-induced alterations in motor cortices, including M2, are strongly implicated in the automatized, compulsive actions [6] that characterize late withdrawal. Recent work from our group has established the importance of cortical pyramidal neurons in M2, particularly those in Layer 2, during intravenous self-administration of cocaine. Using longitudinal in vivo calcium imaging during cocaine self-administration, we demonstrated that M2 cortical pyramidal neuron ensembles dynamically encode cocaine-taking behavior, exhibiting temporal and spatial alterations in their activity across self-administration days and between early vs. late stages within a daily self-administration session [7, 8]. Furthermore, we showed that reducing M2 cortical pyramidal neuron excitability, either pharmacologically or via chemogenetic approaches, significantly attenuates cocaine seeking [9].

Similar to other cortical areas, M2 does not work in isolation. Anatomically, M2 sends dense projections to both the dorsolateral striatum (DLS) and dorsomedial striatum (DMS). These two subregions play complementary roles in drug seeking: the DLS promotes habitual, stimulus-response behaviors, whereas the DMS contributes to goal-directed control [10–14]. Altered excitability of M2 cortical pyramidal neurons and their projections to these striatal targets could therefore bias striatal output toward relapse-related behaviors. While adaptations in medial prefrontal-striatal communication after cocaine exposure have been extensively studied [15, 16], much less is known about how prolonged withdrawal alters M2 projections to the DLS and DMS, and how such reorganization contributes to relapse vulnerability.

To start addressing this knowledge gap, we combined a rat intravenous self-administration model with pathway-selective optogenetic manipulations and ex vivo patch-clamp electrophysiology. Using this approach, we first confirmed cocaine seeking was enhanced on withdrawal day 45, relative to withdrawal day 1, consistent with the incubation of cocaine seeking previously reported by others and by our laboratory [1, 9, 16, 17]. We then assessed intrinsic excitability of M2 cortical pyramidal neurons, tested the behavioral consequences of optogenetic inhibition of eNpHR-expressing M2-DLS *vs*. M2-DMS terminals, and probed the underlying synaptic mechanisms by recording both excitatory and inhibitory responses in striatal medium-sized spiny neurons (MSNs). This integrative strategy allowed us to assess how M2 output across distinct projection regions contributes to relapse-related behaviors and how prolonged withdrawal reshapes its synaptic output.

## Materials and methods

All experiments were conducted in accordance with the United States Public Health Service Guide for Care and Use of Laboratory Animals and were approved by the Institutional Animal Care and Use Committee at Indiana University School of Medicine. Male Sprague-Dawley rats underwent jugular catheterization, M2-targeted AAV injection, and, in some cases, DLS/DMS optic fiber implantation. During the cocaine taking sessions, rats were placed in the self-administration chamber on a fixed ratio 1 reinforcement schedule with the house light on. Active lever presses resulted in a cocaine infusion (0.75 mg/kg over 2–4 s) and the illumination of a CS light above the active lever for 20 s with the house light off. In contrast, the inactive lever presses led to no outcome but were also recorded. During the cocaine seeking test, rats were re-exposed to the operant chamber with the exact same conditions as during cocaine self-administration training, except no cocaine IV delivery was programmed after pressing the previous intravenous delivery-associated lever. To avoid potential carryover effects from repeated extinction testing, each rat assigned to an in vivo cocaine seeking test was evaluated at only a single time point. Rats assigned to ex vivo electrophysiology did not undergo any extinction-seeking test. Additional methodological details are provided in the Supplementary Information.

## Results

### Increased cocaine seeking after a prolonged withdrawal period

**Fig. 1:**
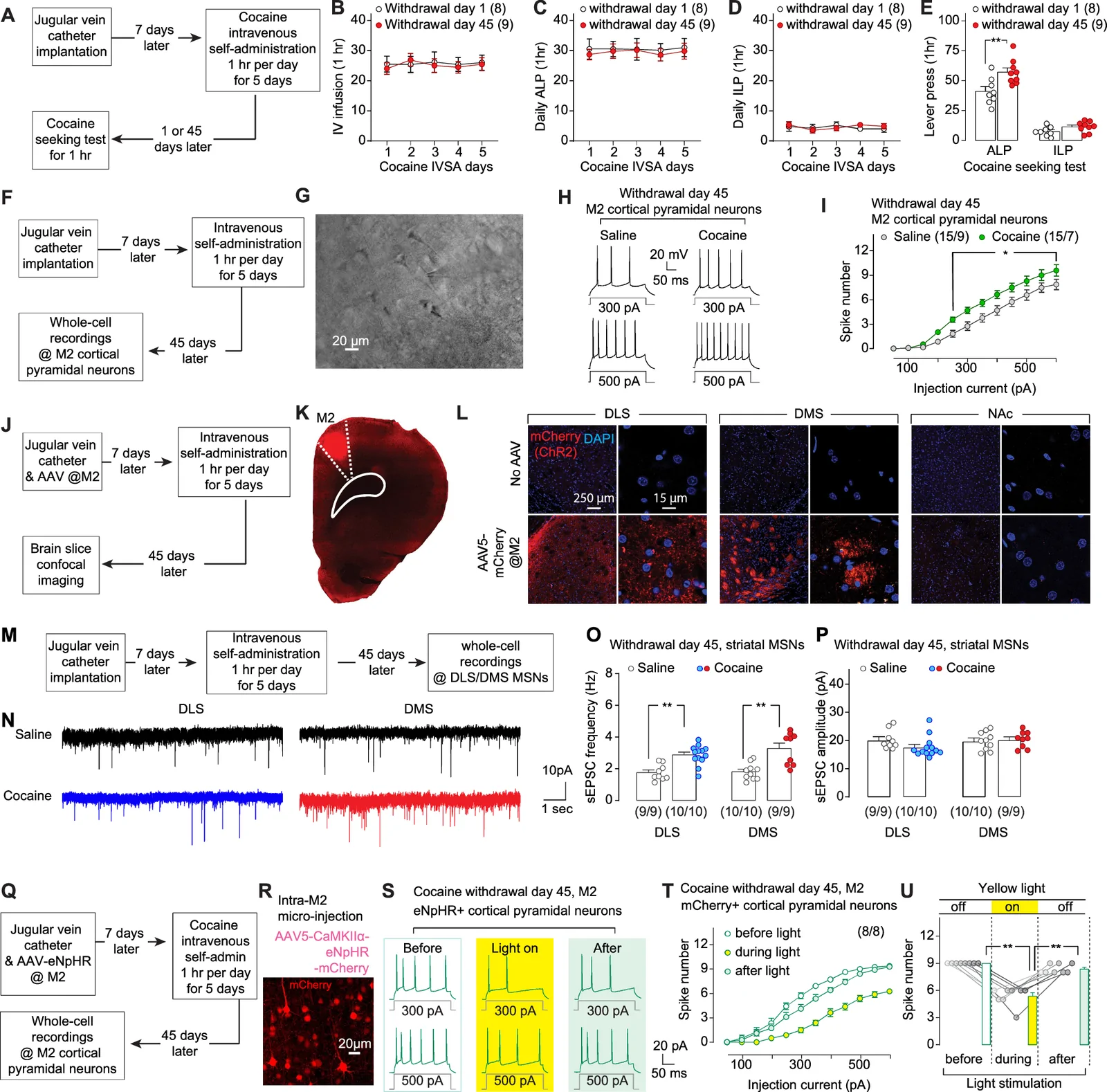
Increased cocaine seeking, M2 hyperexcitability, and enhanced striatal excitatory input on withdrawal day 45. **A** Experimental timeline for (**B**–**E**). No differences were observed in the number of cocaine infusion (**B**), active lever presses (**C**), and inactive lever presses (**D**) during the five 1-hr daily cocaine self-administration sessions in rats pre-assigned for cocaine seeking test on withdrawal day 1 *vs*. 45. **E** More active lever presses, but similar inactive lever presses on withdrawal day 45, relative to withdrawal day 1, after cocaine self-administration. **F** Experimental timeline for (**G**–**I**). **G** Representative DIC images of patching an M2 cortical pyramidal neuron. Example recording traces (**H**) and summary data (**I**) showing more spikes in M2 cortical pyramidal neurons from cocaine rats, relative to those from saline rats, on withdrawal day 45. Experimental timeline (**J**) and confocal images showing the AAV-mCherry expression in the viral injection site (M2) (**K**) and their striatal projections in the DLS and DMS, but not NAc (**L**). **M** Experimental timeline for (**N**–**P**). **N** Representative sEPCS traces of MSNs in the DLS (left) and DMS (right) from rats trained by self-administration of saline (upper) and cocaine (lower). Relative to saline rats, cocaine rats demonstrated higher sEPSC frequency (**O**) and similar amplitude (**P**) in MSNs from both the DLS and DMS on withdrawal day 45. **Q** Experimental timeline for (**R**–**U**). **R** Example confocal image showing the expression of AAV5-CaMKII-eNpHR-mCherry in M2 neurons, most likely the cortical pyramidal neurons. **S**–**U** Example traces (**S**) and summary data showing that optogenetic inhibition reduced the spike number in the mCherry-positive, eNpHR-expressing M2 cortical pyramidal neurons, which was reversed when the optogenetic inhibition was off (**T**, **U**). The number of rats or neurons/rats is provided in parentheses. Each scatter dot represents the value from an individual rat (**E**) or neuron (**O**, **P**, **U**). Data were analyzed by one-way ANOVA with repeated measures on optogenetic inhibitions (**U**), two-way ANOVA with repeated measures on self-administration day (**B**–**D**), on injection current (**I**), on injection current and optogenetic inhibition (**T**), and two-way ANOVA (**E**, **O**, **P**), followed by Bonferroni’s post hoc test. *p < 0.05; **p < 0.01.

Two groups of rats (Fig. 1A) with comparable cocaine-taking histories, reflected by similar numbers of intravenous infusions (Fig. 1B, self-administration day × withdrawal day 1/45 interaction *F*_4,60_ = 1.0, *p* = 0.40; withdrawal day *F*_4,60_ = 1.0, *p* = 0.43; withdrawal day 1/45 *F*_1,15_ = 0.0, *p* = 0.87), active lever presses (Fig. 1C, self-administration day × withdrawal day 1/45 interaction *F*_4,60_ = 0.1, *p* = 0.97; self-administration day *F*_4,60_ = 0.3, *p* = 0.83; withdrawal day 1/45 *F*_1,15_ = 0.1, *p* = 0.74), and inactive lever presses (Fig. 1D, self-administration day × withdrawal day 1/45 interaction *F*_4,60_ = 1.3, *p* = 0.26; self-administration day *F*_4,60_ = 1.0, *p* = 0.44; withdrawal day 1/45 *F*_1,15_ = 0.0, *p* = 0.86) during five 1-h daily self-administration training sessions, were tested for cocaine seeking on either withdrawal day 1 or 45. A significantly higher number of active lever presses was observed on withdrawal day 45, relative to withdrawal day 1, while the number of inactive lever presses remained unchanged between the two time points (Fig. 1E, active/inactive lever press × withdrawal day 1/45 interaction *F*_1,15_ = 6.0, *p* = 0.03; active/inactive lever press *F*_1,15_ = 251.0, *p* < 0.01; withdrawal day 1/45 *F*_1,15_ = 1.5, *p* = 0.22). These findings successfully replicate the elevated drug-seeking behavior observed after prolonged withdrawal, as previously reported as incubation of drug seeking by our group and others [9, 16, 18].

### M2 cortical pyramidal neuron excitability and its excitatory synaptic projection to the dorsal striatum

Using whole-cell patch clamp recordings, we assessed the intrinsic excitability of M2 layer 2 cortical pyramidal neurons on withdrawal day 45 following saline or cocaine self-administration (Fig. 1F, G). We observed a significant increase in spike number in M2 cortical pyramidal neurons from cocaine-exposed rats compared to saline controls (Fig. 1H, I, saline/cocaine × injection current interaction *F*_11,308_ = 3.5, *p* < 0.01; saline/cocaine *F*_1,28_ = 6.2, *p* = 0.02; injection current *F*_11,308_ = 263.1, *p* < 0.01). Together with our recent findings showing no detectable differences in M2 cortical pyramidal neuron excitability (1) 1 day after saline vs. cocaine self-administration, and (2) 1 vs. 45 days after sucrose self-administration, as well as evidence that pharmacological or chemogenetic attenuation of M2 pyramidal neuron excitability reduces cocaine seeking at later withdrawal stages [9, 19], our data indicate that M2 cortical hyperexcitability could be a contributing factor to elevated cocaine-seeking behavior.

Confocal imaging of brain slices from rats bilaterally injected with AAV-CaMKIIα-mCherry in M2 revealed that M2 projections primarily target neurons in the dorsolateral striatum (DLS) and dorsomedial striatum (DMS), but not the nucleus accumbens (NAc) (Fig. 1J–L). We then recorded spontaneous excitatory postsynaptic currents (sEPSCs) in the M2-striatal projections (Fig. 1M, N) and found that cocaine self-administration increased sEPSC frequency (Fig. 1O, saline/cocaine × DLS/DMS interaction *F*_1,37_ = 0.6, *p* = 0.43; saline/cocaine *F*_1,37_ = 36.3, *p* < 0.01; DLS/DMS *F*_1,37_ = 1.1, *p* = 0.30), but not amplitude (Fig. 1P, saline/cocaine × DLS/DMS interaction *F*_1,37_ = 2.1, *p* = 0.15; saline/cocaine *F*_1,37_ = 1.0, *p* = 0.32; DLS/DMS *F*_1,37_ = 0.3, *p* = 0.28), in both M2-DLS and M2-DMS projections on withdrawal day 45 relative to saline controls. These findings suggest a potential enhancement of presynaptic glutamate release, possibly driven by the observed hyperexcitability of M2 cortical pyramidal neurons' projection to both DLS and DMS. Since the focus of the current study was to determine the role of M2-striatal projections in the incubation of drug seeking, circuit-specific synaptic transmission was evaluated on WD45, when incubation of cocaine seeking was robustly detected. We acknowledge that assessing M2 circuit-specific synaptic transmission at an earlier withdrawal time point (e.g., WD1) would strengthen the temporal association between synaptic adaptations and the progression of cocaine-seeking behavior. Because early withdrawal was not examined here, similarity to previously reported somatic adaptations should be interpreted cautiously.

Another group of rats received bilateral intra-M2 microinjections of AAV5-CaMKIIα-eNpHR3.0-mCherry in the same surgical session of jugular vein catheter implantation. The intrinsic excitability of mCherry-positive M2 cortical pyramidal neurons was evaluated before, during, and after yellow light (*λ* = 590 nm) inhibition on withdrawal day 45 following cocaine self-administration (Fig. 1Q, R). We found that the excitability of these mCherry-positive cortical pyramidal neurons was transiently suppressed during optogenetic inhibition (Fig. 1S–U; data from 50-600 pA injection currents in T, light on/off × injection current interaction *F*_2,15_ = 243.5, *p* < 0.01; light on/off *F*_2,13_ = 116.2, *p* < 0.01; injection current *F*_4,28_ = 14.1, *p* < 0.01; data from 400 pA injection current in U, *F*_1,7_ = 601.0, *p* < 0.01), indicating that optogenetic inhibition effectively attenuated the hyperexcitability of eNpHR-expressing M2 cortical pyramidal neurons in cocaine-exposed rats. This led us to investigate whether the increased cocaine seeking and the frequency of sEPSCs in the DLS/DMS on withdrawal day 45 could also be modulated by optogenetic inhibition at the eNpHR-expressing M2-DLS/DMS projections, as addressed in the following experiments.

### Effects of optogenetic inhibition at the M2-DLS/DMS projections on cocaine seeking behaviors on withdrawal day 45

**Fig. 2:**
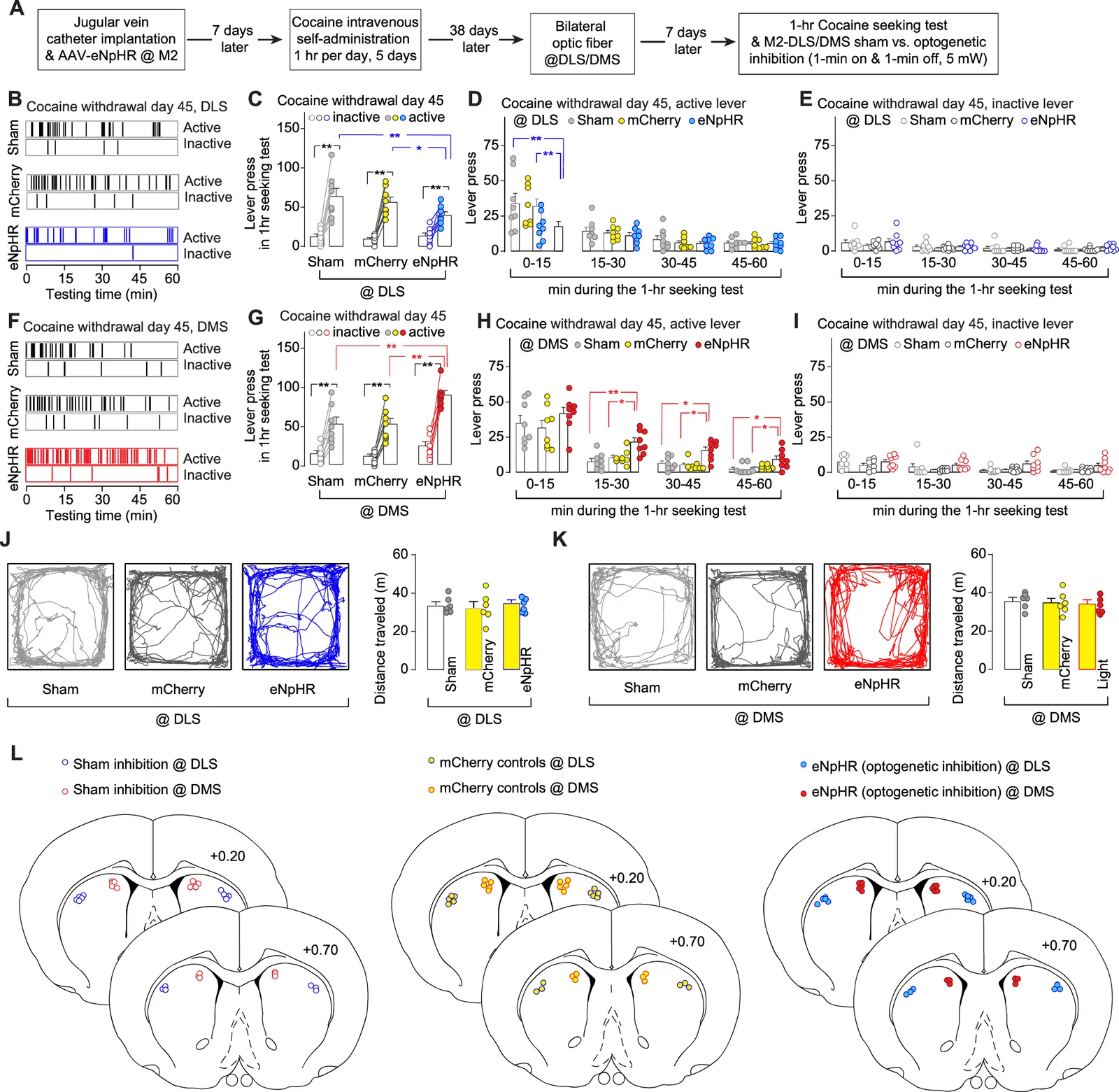
Effects of in vivo 1-min on/1-min off optogenetic inhibition of M2-striatal projections on withdrawal day 45 cocaine seeking behaviors. **A** Experimental timeline. Representative time course plots of cocaine seeking behavior (**B**, top, sham light; middle, mCherry control; bottom, optogenetic-inhibition) and summarized data showing that, relative to sham and mCherry controls, optogenetic inhibition at the eNpHR-expressing M2-DLS projection reduced the number of active lever presses, but not inactive lever presses, during the cocaine seeking test on withdrawal day 45 (**C**). Specifically, the reduction in active lever presses was primarily detected during the first 15 min of the 1-hr seeking test (**D**), while inactive lever presses remained unaffected throughout the seeking test (**E**). Representative time course plots of cocaine seeking behavior (**F**, top, sham light; middle, mCherry control; bottom, optogenetic-inhibition) and summarized data showing that, relative to sham and mCherry controls, optogenetic inhibition at the eNpHR-expressing M2-DMS projection increased the number of active lever presses, but not inactive lever presses, during the cocaine seeking test on withdrawal day 45 (**G**). Specifically, the increase in active lever presses was primarily detected after the first 15 min of the 1-h seeking test (**H**), while inactive lever presses remained unaffected throughout the seeking test (**I**). Representative travel maps and summarized data showing that optogenetic light inhibitions at either the M2-DLS (**J**) or M2-DMS (**K**) projections did not affect the general locomotion in the open field test. **L** Diagrams showing the bilateral optic fiber tips at the DLS (blue) and DMS (red) when the sham (open circles) and optogenetic (solid circles) inhibitions were applied on withdrawal day 45 after cocaine taking sessions. Data were collected from 8 rats per group in (**A**–**I**). **L** and 6 rats per group in (**J**, **K**) and then analyzed by two-way ANOVA with repeated measures on active/inactive lever presses (**C**, **G**) or 15-min time slot (**D**, **E**, **H**, **I**), and one-way ANOVA (**J**, **K**), followed by Bonferroni’s post hoc test. *p < 0.05; **p < 0.01. Black asterisks indicate within-subject comparisons, and blue or red asterisks indicate between-subject comparisons.

As shown in Fig. 2A, rats expressing eNpHR at M2 projection terminals received bilateral yellow light inhibition (*λ* = 590 nm, 5 mW per site, 1 min on and 1 min off) during the cocaine seeking test on withdrawal day 45 following self-administration training. Two control groups were tested in parallel: (1) sham controls, which received low-intensity light stimulation (1% of full power; 0.05 mW per site) during the 1-min light-on epochs in eNpHR-expressing M2-DLS or M2-DMS projections in rats 45 days after cocaine self-administration; and (2) mCherry controls, which received full-intensity optogenetic inhibition (5 mW per site) during the cocaine-seeking test in rats with intra-M2 injection of AAV5-CaMKIIα-mCherry, lacking eNpHR expression, in M2-DLS or M2-DMS projections. We found that, relative to both control groups, optogenetic inhibition of the eNpHR-expressing M2-DLS projection reduced the number of active lever presses during the cocaine-seeking test (Fig. 2B, C, L, active/inactive lever press × sham/ mCherry/eNpHR interaction *F*_2,21_ = 3.6, *p* = 0.04; active/inactive lever press *F*_1,21_ = 104.0, *p* < 0.01; sham/mCherry/eNpHR *F*_2,21_ = 1.7, *p* = 0.20). This reduction was most pronounced during the first 15 min of the 1-hr seeking session (Fig. 2D, 15-min slot × sham/mCherry/eNpHR interaction *F*_6,63_ = 2.3, *p* = 0.047; 15-min slot *F*_3,63_ = 36.2, *p* < 0.01; sham/mCherry/eNpHR *F*_2,21_ = 2.7, *p* = 0.11). In contrast, inactive lever press number was unaffected by optogenetic inhibition across the session (Fig. 2E, 15-min slot × sham/mCherry/eNpHR interaction *F*_6,63_ = 0.4, *p* = 0.87; 15-min slot *F*_3,63_ = 9.4, *p* < 0.01; sham/mCherry/eNpHR *F*_2,21_ = 0.5, *p* = 0.61). Interestingly, the same light inhibition protocol applied to the M2-DMS projection enhanced cocaine-seeking behavior, i.e., active lever presses (Fig. 2G, active/inactive lever press × sham/mCherry/eNpHR interaction *F*_2,21_ = 4.4, *p* = 0.02; active/inactive lever press *F*_1,21_ = 139.7, *p* < 0.01; sham/mCherry/eNpHR *F*_2,21_ = 9.2, *p* < 0.01), with the effects becoming more pronounced after the first 15 min of the session (Fig. 2H, 15-min slot × sham/mCherry/eNpHR interaction *F*_6,63_ = 0.5, *p* = 0.82; 15-min slot *F*_3,63_ = 70.5, p < 0.01; sham/mCherry/eNpHR *F*_2,21_ = 8.8, *p* < 0.01). Similar to the DLS group, inactive lever presses remained unaffected throughout the 1-hr seeking test after optical inhibition on M2-DMS projection (Fig. 2I, 15-min slot × sham/mCherry/eNpHR interaction *F*_6,63_ = 1.0, *p* = 0.40; 15-min slot *F*_3,63_ = 8.7, *p* < 0.01; sham/mCherry/eNpHR *F*_2,21_ = 3.1, *p* = 0.07).

To determine whether this manipulation impacted general locomotor activity, we conducted a 5-min open field test under the same optogenetic inhibition or two different control conditions a couple of days after the cocaine seeking test. No differences in total distance traveled were observed between the optogenetic inhibition and sham groups, regardless of whether the inhibition targeted the M2-DLS (Fig. 2J, *F*_2,15_ = 0.3, *p* = 0.76) or M2-DMS (Fig. 2K, *F*_2,15_ = 0.1, *p* = 0.87) projections. These results suggest that optogenetic inhibition of distinct eNpHR-expressing M2 output pathways can differentially regulate cocaine-seeking behavior without altering overall locomotion. The following ex vivo experiments were aimed at exploring the synaptic effects of this circuit-specific optogenetic inhibition.

### Synaptic effects of optogenetic inhibition at the M2-DLS/DMS projections on withdrawal day 45 after cocaine intravenous self-administration

**Fig. 3:**
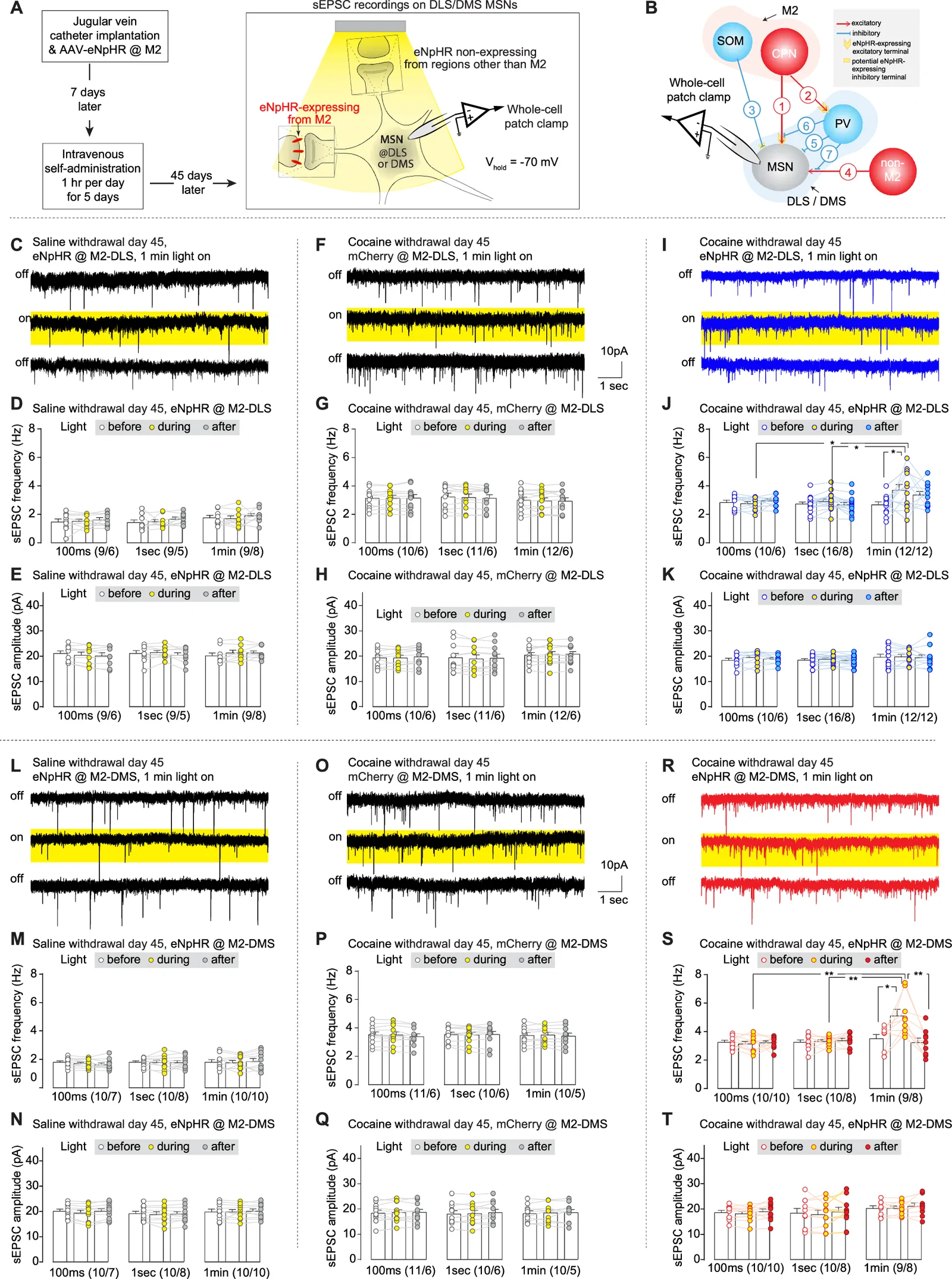
Effects of optogenetic inhibition of M2-striatal projections with varying durations on MSN sEPSCs, on withdrawal day 45 in saline *vs*. cocaine rats. **A** Experimental timeline and diagram of the synaptic components involved in sEPSC recordings in DLS/DMS MSNs for data collection in Figs. 3, 4 and S1. **B** Hypothetical corticostriatal and local circuit inputs to striatal MSNs. M2 cortical pyramidal neurons provide glutamatergic input directly to MSNs (1) and to local interneurons (PV, 2), as well as inhibitory projections to MSNs (3). MSNs also receive excitatory inputs from non-M2 sources such as anterior cingulate, insular cortex, hippocampus, thalamus, and amygdala (4). Local interneurons inhibit MSNs (5) and suppress glutamate release from M2 (6) and non-M2 (7) terminals. Whole-cell patch-clamp recordings were performed on DLS/DMS MSNs, with trans-membrane voltage-clamped at -70 mV to record sEPSCs and at +10 mV to record sIPSCs. Example sEPSC traces (**C**) and the summary data showing optogenetic inhibition at the eNpHR-expressing M2-DLS projection did not affect the sEPSC frequency (**D**) or amplitude (**E**) in DLS MSNs on withdrawal day 45 in saline rats. **F**–**H** Example sEPSC traces (**F**) and the summary data showing light application at the mCherry-expressing M2-DLS projection did not affect the sEPSC frequency (**D**) or amplitude (**E**) in DLS MSNs on withdrawal day 45 in cocaine rats. Example sEPSC traces (**I**) and the summary data showing optogenetic inhibition for 1 min, but not 100 ms or 1 s, at the eNpHR-expressing M2-DLS projection increased the sEPSC frequency (**J**) but not amplitude (**K**) in DLS MSNs on withdrawal day 45 in cocaine rats. Example sEPSC traces (**L**) and the summary data showing optogenetic inhibition at the eNpHR-expressing M2-DMS projection did not affect the sEPSC frequency (**M**) or amplitude (**N**) in DMS MSNs on withdrawal day 45 in saline rats. Example sEPSC traces (**O**) and the summary data showing light application at the mCherry-expressing M2-DMS projection did not affect the sEPSC frequency (**P**) or amplitude (**Q**) in DMS MSNs on withdrawal day 45 in cocaine rats. Example sEPSC traces (**R**) and the summary data showing optogenetic inhibition for 1 min, but not 100 ms or 1 s, at the eNpHR-expressing M2-DMS projection affected the sEPSC frequency (**S**) but not amplitude (**T**) in DMS MSNs on withdrawal day 45 in cocaine rats. The number of neurons/rats is available in (**D**, **E**, **G**, **H**, **J**, **K**, **M**, **N**, **P**, **Q**, **S**, **T**). Data were analyzed by two-way ANOVA with repeated measures on the light on/off condition, followed by Bonferroni’s post hoc test. *p < 0.05; **p < 0.01.

Effects of three different durations of optogenetic inhibition at mCherry only or eNpHR-expressing M2-DLS or M2-DMS projections were evaluated by recording sEPSCs from MSNs in brain slices prepared 45 days after cocaine self-administration (Fig. 3A). Whole-cell patch-clamp recordings revealed that in cocaine-exposed rats, yellow light inhibition for 1 min, but not 100 ms or 1 s, significantly increased the frequency of sEPSCs in MSNs when either M2-DLS (Fig. 3I, J, light duration × light on/off interaction *F*_4,69_ = 2.9, *p* = 0.04; light duration *F*_2,35_ = 3.3, *p* = 0.048; light on/off *F*_2,69_ = 3.3, *p* = 0.04) or M2-DMS (Fig. 3R, S, light duration × light on/off interaction *F*_4,48_ = 8.1, *p* < 0.01; light duration *F*_2,26_ = 6.0, *p* = 0.01; light on/off *F*_2,48_ = 7.0, *p* < 0.01) projections were targeted, although no changes of sEPSC amplitude were detected in M2-DLS (Fig. 3K, light duration × light on/off interaction *F*_4,68_ = 0.6, *p* = 0.66; light duration *F*_2,35_ = 0.6, *p* = 0.56; light on/off *F*_2,68_ = 2.3, *p* = 0.11) or M2-DMS (Fig. 3T, light duration × light on/off interaction *F*_3,43_ = 0.1, *p* = 0.97; light duration *F*_2,26_ = 0.9, *p* = 0.42; light on/off *F*_2,43_ = 2.4, *p* = 0.12). In eNpHR saline controls, no changes in sEPSC frequency were detected when optogenetic inhibition was applied to either M2-DLS (Fig. 3C, D, light duration × light on/off interaction *F*_4,44_ = 0.1, *p* = 0.98; light duration *F*_2,24_ = 1.2, *p* = 0.31; light on/off *F*_2,44_ = 1.8, *p* = 0.18) or M2-DMS (Fig. 3L, M, light duration × light on/off interaction *F*_4,49_ = 0.8, *p* = 0.55; light duration *F*_2,27_ = 0.2, *p* = 0.84; light on/off *F*_2,49_ = 0.5, *p* = 0.59) projection, nor were there changes in sEPSC amplitude when optogenetic inhibition was applied to M2-DLS (Fig. 3E, light duration × light on/off interaction *F*_4,47_ = 1.8, *p* = 0.14; light duration *F*_2,24_ = 0.1, *p* = 0.94; light on/off *F*_2,48_ = 1.4, *p* = 0.25) or M2-DMS (Fig. 3N, light duration × light on/off interaction *F*_4,43_ = 0.9, *p* = 0.48; light duration *F*_2,27_ = 0.3, *p* = 0.74; light on/off *F*_2,43_ = 2.6, *p* = 0.10). In mCherry cocaine controls, no changes in sEPSC frequency were detected when optogenetic inhibition was applied to M2-DLS (Fig. 3F, G, light duration × light on/off interaction *F*_3,50_ = 8.1, *p* = 0.50; light duration *F*_2,31_ = 0.3, *p* = 0.74; light on/off *F*_2,50_ = 0.7, *p* = 0.49) or M2-DMS (Fig. 3O, P, light duration × light on/off interaction *F*_3,42_ = 1.0, *p* = 0.40; light duration *F*_2,29_ < 0.1, *p* = 0.97; light on/off *F*_1,42_ = 1.9, *p* = 0.17), nor were there changes in sEPSC amplitude when optogenetic inhibition was applied to M2-DLS (Fig. 3H, light duration × light on/off interaction *F*_3,52_ = 0.9, *p* = 0.45; light duration *F*_2,31_ = 0.4, *p* = 0.69; light on/off *F*_2,52_ = 1.4, *p* = 0.25) or M2-DMS (Fig. 3Q, light duration × light on/off interaction *F*_3,42_ = 0.1, *p* = 0.97; light duration *F*_2,29_ < 0.1, *p* = 0.99; light on/off *F*_1,42_ = 1.1, *p* = 0.32). Thus, this circuit-specific, optogenetic inhibition-induced increase in sEPSC frequency in cocaine rats was unexpected and cannot be attributed to light stimulation itself. To further characterize this effect, we quantified sEPSC events in 10-s time bins. Consistent with the overall findings, 1-min inhibition of eNpHR-expressing M2-DLS (Fig. S1A, B) or M2-DMS terminals (Fig. S1C, D) in cocaine-treated rats selectively increased sEPSC frequency, but not amplitude. The frequency increase emerged immediately after light onset, predominantly within the first 10-s window. Using the M2-DLS pathway as the example, the effect was duration-dependent: 100-ms pulses increased frequency by 0.2 Hz (7%), from 2.3 Hz pre-inhibition to 2.5 Hz; 1-s inhibition produced a 0.6 Hz (19%) increase, from 2.9 Hz to 3.5 Hz; and 1-min inhibition yielded a 2.3 Hz (109%) increase, from 2.1 Hz to 4.4 Hz. These results suggest that while optogenetic inhibition was applied specifically to eNpHR-expressing M2 projections, the observed duration-dependent increase in sEPSC frequency reflects an overall enhancement of excitatory drive onto MSNs as indicated in Fig. 3B, rather than a strictly circuit-restricted effect, and is unlikely to be attributable to light-induced artifacts.

**Fig. 4:**
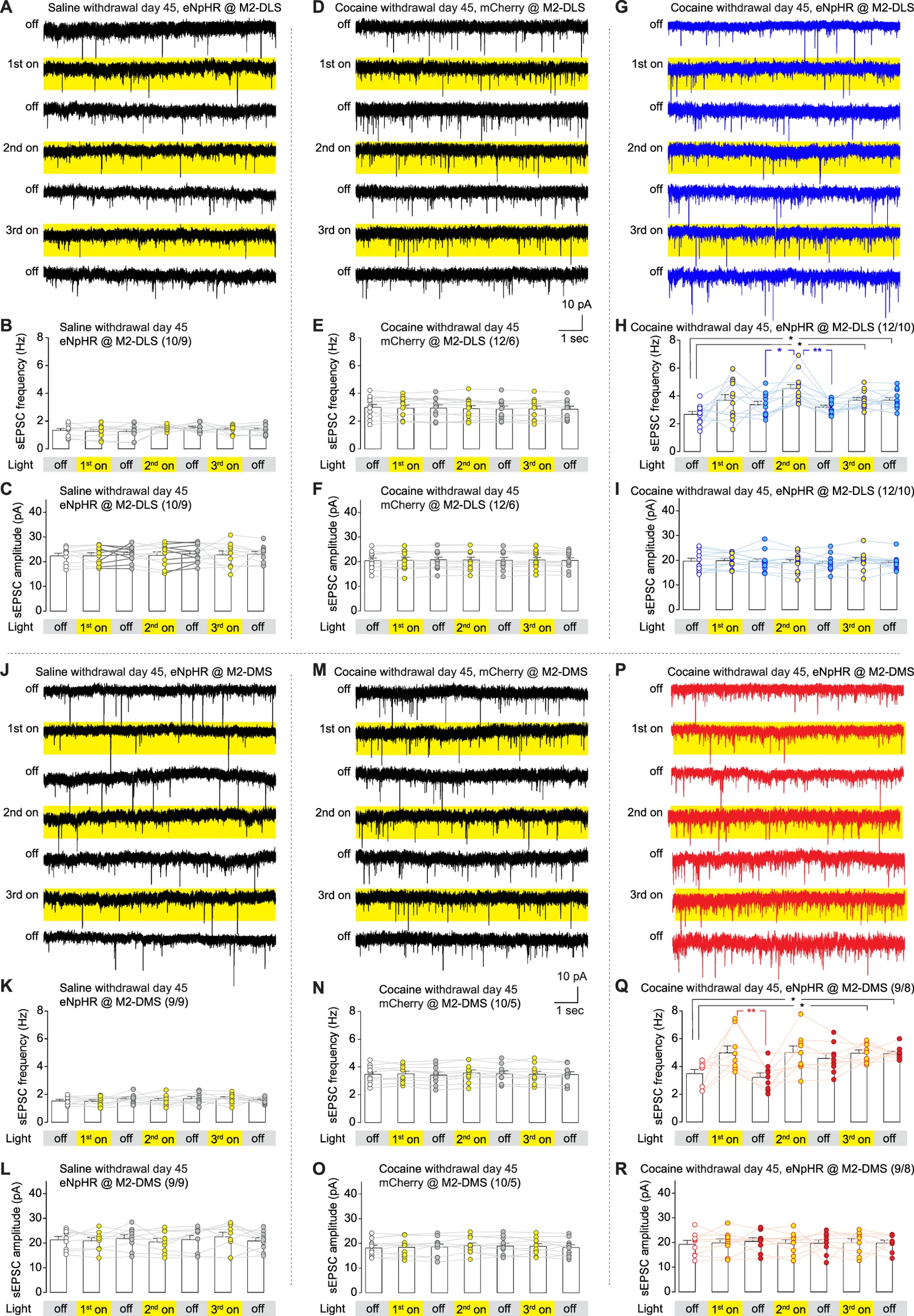
Effects of repeated 1-min on/1-min off optogenetic inhibition of M2-striatal projections on MSN sEPSCs on withdrawal day 45 in saline or cocaine rats. Example sEPSC traces (**A**) and the summary data showing repeated 1-min on/1-min off optogenetic inhibition at the eNpHR-expressing M2-DLS projection did not affect the sEPSC frequency (**B**) or amplitude (**C**) in DLS MSNs on withdrawal day 45 in saline rats. Example sEPSC traces (**D**) and the summary data showing repeated 1-min on/1-min off light application at the mCherry-expressing M2-DLS projection did not affect the sEPSC frequency (**E**) or amplitude (**F**) in DLS MSNs on withdrawal day 45 in cocaine rats. Example sEPSC traces (**G**) and the summary data showing repeated 1-min on/1-min off optogenetic inhibition at the eNpHR-expressing M2-DLS projection affected the sEPSC frequency (**H**) but not amplitude (**I**) in DLS MSNs on withdrawal day 45 in cocaine rats. Example sEPSC traces (**J**) and the summary data showing repeated 1-min on/1-min off optogenetic inhibition at the eNpHR-expressing M2-DMS projection did not affect the sEPSC frequency (**K**) or amplitude (**L**) in DMS MSNs on withdrawal day 45 in saline rats. **M–O** Example sEPSC traces (**M**) and the summary data showing repeated 1-min on/1-min off light application at the mCherry-expressing M2-DMS projection did not affect the sEPSC frequency (**O**) or amplitude (**P**) in DMS MSNs on withdrawal day 45 in cocaine rats. Example sEPSC traces (**P**) and the summary data showing repeated 1-min on/1-min off optogenetic inhibition at the eNpHR-expressing M2-DMS projection affected the sEPSC frequency (**Q**) but not amplitude (**R**) in DMS MSNs on withdrawal day 45 in cocaine rats. The number of neurons/rats is available in (**B**, **C**, **E**, **F**, **H**, **I**, **K**, **L**, **N**, **O**, **Q**, **R**). Data were analyzed by one-way ANOVA with repeated measures on light on/off condition, followed by Bonferroni’s post hoc test. *p < 0.05, **p < 0.01, between adjacent 1-min time blocks (blue for DLS and red for DMS), or relative to the first 1-min light-light off time block (black).

We then examined the effects of repeated 1-min optogenetic inhibitions delivered to the M2-DLS/DMS projections, using the same temporal pattern as in the in vivo evaluations. On withdrawal day 45 following saline self-administration training, optogenetic inhibitions had no detectable effects on sEPSCs recorded from MSNs: neither sEPSC frequency nor amplitude was altered in the DLS (Fig. 4A–C: frequency in **B**, *F*_3,23_ = 1.0, *p* = 0.43; amplitude in **C**, *F*_2,21_ = 0.1, *p* = 0.90) or in the DMS (Fig. 4J–L: frequency in **K**, *F*_3,27_ = 0.6, *p* = 0.61; amplitude in **L**, F_3,24_ = 1.0, *p* = 0.40). Similarly, in cocaine mCherry controls, light inhibitions had no detectable impact on sEPSC frequency or amplitude in either DLS MSNs (Fig. 4D–F: frequency in **E**, *F*_3,33_ = 1.0, *p* = 0.42; amplitude in **F**, *F*_2,23_ = 1.0, *p* = 0.41) or DMS MSNs (Fig. 4M-O: frequency in **N**, *F*_3,25_ = 1.2, *p* = 0.34; amplitude in **O**, *F*_3,28_ = 1.2, *p* = 0.32). In contrast, on withdrawal day 45 following cocaine self-administration in eNpHR-expressing rats, the same repeated 1-min inhibitions selectively increased the frequency, but not the amplitude, of sEPSCs in MSNs from both the DLS (Fig. 4G–I: frequency in **H**, *F*_3,34_ = 5.5, *p* < 0.01; amplitude in **I**, *F*_3,37_ = 0.8, *p* = 0.54) and DMS (Fig. 4P–R: frequency in **Q**, *F*_3,23_ = 6.5, *p* < 0.01; amplitude in **R**, *F*_3,24_ = 0.1, *p* = 0.97). Analysis of the inhibition dynamics showed that sEPSC frequency in both regions initially fluctuated between light-on and light-off periods during the first and second inhibition cycles. By the third cycle, however, these fluctuations diminished, and the frequency increase stabilized, with no further differences between light-on and light-off periods. Together, these results indicate that repeated circuit-specific inhibition of M2 projections leads to a stabilization of excitatory drive in both the DLS and DMS after several light-on/light-off cycles.

**Fig. 5:**
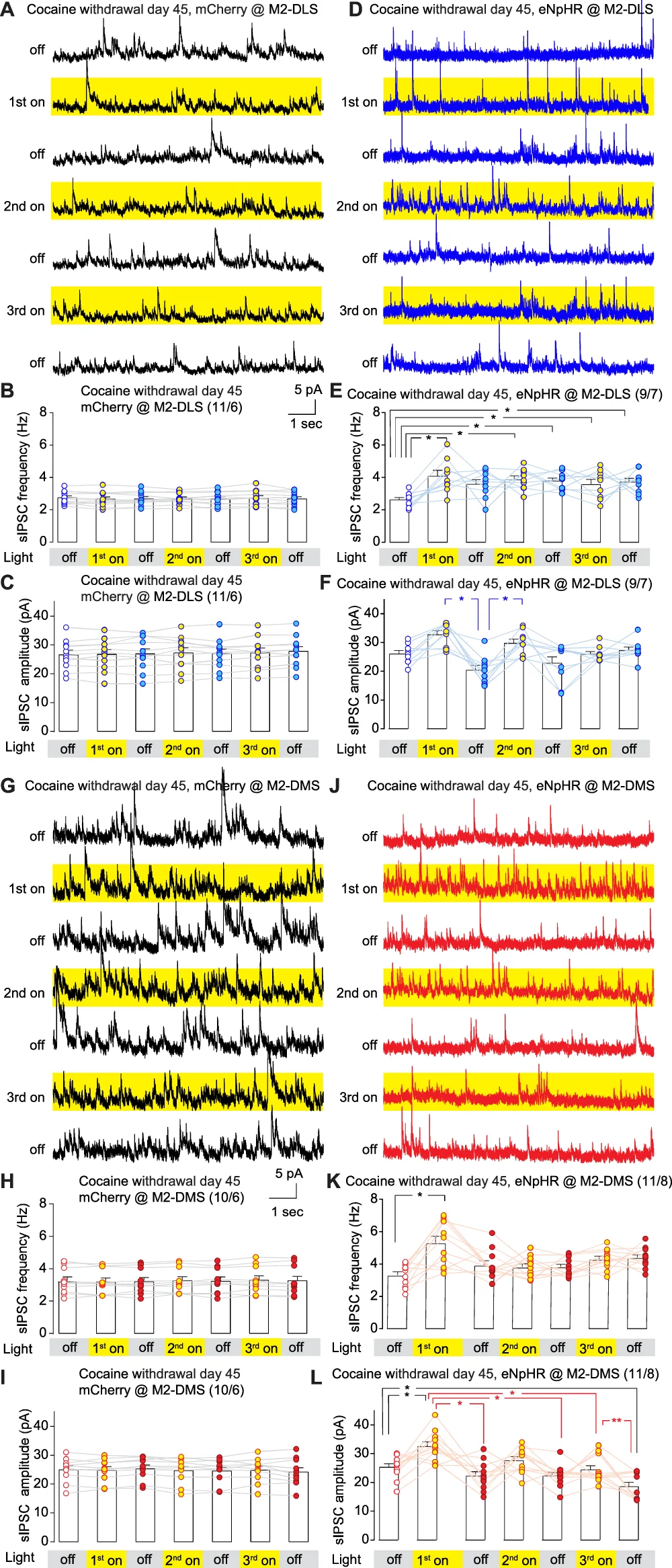
Effects of repeated 1-min on/1-min off optogenetic inhibition of M2-striatal projections on MSN sIPSCs on withdrawal day 45 in cocaine rats. Example sIPSC traces (**A**) and the summary data showing repeated 1-min on/1-min off light application at the mCherry-expressing M2-DLS projection did not affect the sIPSC frequency (**B**) or amplitude (**C**) in DLS MSNs on withdrawal day 45 in cocaine rats. Example sIPSC traces (**D**) and the summary data showing repeated 1-min on/1-min off optogenetic inhibitions at the eNpHR-expressing M2-DLS projection affected the sIPSC frequency (**E**) and amplitude (**F**) in DLS MSNs on withdrawal day 45 in cocaine rats. Example sIPSC traces (**G**) and the summary data showing repeated 1-min on/1-min off light application at the mCherry-expressing M2-DMS projection did not affect the sIPSC frequency (**H**) or amplitude (**I**) in DMS MSNs on withdrawal day 45 in cocaine rats. Example sIPSC traces (**J**) and the summary data showing repeated 1-min on/1-min off optogenetic inhibitions at the eNpHR-expressing M2-DMS projection affected the sIPSC frequency (**K**) and amplitude (**L**) in DMS MSNs on withdrawal day 45 in cocaine rats. The number of neurons/rats is available in (**B**, **C**, **E**, **F**, **H**, **I**, **K**, **L**). Data were analyzed by one-way ANOVA with repeated measures on light on/off condition, followed by Bonferroni’s post hoc test. *p < 0.05, **p < 0.01, between adjacent 1-min time blocks (blue for DLS and red for DMS) or relative to the first 1-min light-light off time block (black).

Given the unexpected increase in sEPSC frequency induced by optogenetic inhibition of eNpHR-expressing M2-DLS and M2-DMS projections, we extended our examinations to inhibitory transmission by recording spontaneous inhibitory postsynaptic currents (sIPSCs) from MSNs in the DLS and DMS 45 days after cocaine self-administration. 1-min optogenetic inhibition of the eNpHR-expressing M2-DLS pathway significantly increased sIPSC frequency, and this enhancement persisted across inhibition epochs regardless of subsequent light on/off conditions after the 2^nd^ light on session (Fig. 5D, E, *F*_4,29_ = 3.5, *p* = 0.02). In contrast, 1-min optogenetic inhibition of the eNpHR-expressing M2-DMS pathway increased sIPSC frequency only during the first light-on period, with no further responses during subsequent inhibitions (Fig. 5J, K, *F*_3,35_ = 6.6, *p* < 0.01). For sIPSC amplitude, distinct patterns emerged between the two eNpHR-expressing pathways. In M2-DMS, optogenetic inhibition initially increased amplitude during the first light-on session but then progressively shifted toward attenuation, relative to baseline before the first light-on session (Fig. 5L, *F*_4,38_ = 10.7, *p* < 0.01). In M2-DLS, optogenetic inhibition produced fluctuations in sIPSC amplitude across the first two light-on sessions but without a sustained cumulative effect (Fig. 5F, *F*_4,29_ = 7.9, *p* < 0.01). In the mCherry cocaine rats, no changes of either frequency or amplitude of sEPSCs were detected when the 1-min optogenetic inhibition was applied to the M2-DLS (Fig. 5A-C: frequency in **B**, *F*_4,38_ = 1.0, i = 0.41; amplitude in **C**, *F*_3,30_ = 1.8, *p* = 0.17) or M2-DMS (Fig. 5G–I: frequency in **H**, *F*_3,34_ = 1.2, *p* = 0.33; amplitude in **I**, *F*_2,16_ = 1.0, *p* = 0.36). These results suggest that on cocaine withdrawal day 45, M2 input may engage inhibitory circuitry in the DLS in a more persistent manner, potentially involving enhanced inhibitory control, whereas in the DMS, this engagement appears transient. While this pattern is consistent with differential inhibitory regulation across striatal subregions, the underlying mechanisms were not directly tested. In addition to possible postsynaptic changes, these effects may also reflect differences in the recruitment of inhibitory interneurons or presynaptic mechanisms, such as alterations in GABA release probability, vesicle content, or the number of functional presynaptic terminals.

## Discussion

In this study, we show that M2-striatal projections undergo synaptic adaptations that differentially influence cocaine seeking. Following cocaine self-administration, M2 neurons became hyperexcitable, and optogenetic inhibition of their eNpHR-expressing terminals in the DLS versus the DMS produced opposite effects on cocaine-seeking behavior, highlighting pathway-specific roles in relapse vulnerability. Notably, optogenetic inhibition of M2 inputs to the striatum unexpectedly enhanced both excitatory and inhibitory synaptic transmission in the DLS and DMS. To interpret these findings, it is necessary to consider the organization of M2 inputs within striatal microcircuits, which we outline in the following section, “Unexpected facilitation of excitatory synaptic activity by optogenetic inhibition”.

### M2 Hyperexcitability and striatal projection in the cocaine seeking model

The observed increase in M2 cortical pyramidal neuron excitability after cocaine self-administration aligns with prior evidence that cocaine withdrawal enhances intrinsic excitability in prefrontal cortical neurons [9, 20]. Extending this work, we found that prolonged withdrawal was associated with increased spontaneous excitatory synaptic activity in both the DLS and DMS. Because sEPSC frequency reflects integrated excitatory input from multiple presynaptic sources, this enhancement does not provide circuit-specific evidence for increased glutamate release from M2 terminals alone. Instead, it is consistent with a broader increase in excitatory drive within corticostriatal networks during abstinence, potentially biasing striatal processing toward drug-seeking behaviors. Importantly, the divergent behavioral effects of inhibiting M2-DLS vs. M2-DMS projections indicate that increased excitatory drive alone is insufficient to explain relapse-related behavior.

### Unexpected facilitation of excitatory synaptic activity by optogenetic inhibition

A surprising finding was that prolonged optogenetic inhibition (1-min light epochs) of eNpHR-expressing M2 terminals increased sEPSC frequency in both the DLS and DMS after cocaine self-administration. This effect was absent in saline controls and mCherry-expressing cocaine controls, was strongly duration dependent, and emerged rapidly during the initial light-on period. Repeated cycles of inhibition further stabilized the increase, such that enhanced sEPSC frequency persisted even during subsequent light-off epochs. The absence of accompanying amplitude changes suggests that this effect is unlikely to reflect postsynaptic scaling and instead points to presynaptic or network-level adaptations.

Two points are critical for interpreting this paradoxical facilitation. First, MSNs integrate convergent excitatory and inhibitory inputs from multiple cortical and subcortical sources. Second, the mechanisms underlying the increased sEPSC frequency were not directly tested in the present study and are therefore considered hypothesis-generating.

As shown in Fig. 3B, M2 cortical pyramidal neurons provide direct glutamatergic input to MSNs (pathway 1) as well as to Parvalbumin (PV)-expressing interneurons (pathway 2) [21], and also give rise to long-range inhibitory projections from somatostatin (SOM)-expressing neurons to striatum (pathway 3) [22–24]. In addition, MSNs receive robust excitatory drive from non-M2 sources (pathway 4), including anterior cingulate, insular cortex, hippocampus, thalamus, and amygdala [25–28]. Local PV interneurons further sculpt excitatory drive by providing feedforward inhibition of MSNs (pathway 5) and feedback regulation of presynaptic glutamate release from both M2 (pathway 6) and non-M2 terminals (pathway 7) [28].

CaMKIIα is highly enriched in cortical pyramidal neurons relative to the inhibitory neurons, and has been widely used as a promoter driver for pyramidal neuron-biased viral expression [29]. However, its specificity for excitatory neurons is not absolute, as CaMKIIα-driven AAV expression has also been reported in subsets of inhibitory neurons [30]. Thus, we propose that intra-M2 delivery of AAV5-CaMKIIα-eNpHR-mCherry resulted in the eNpHR expression predominantly at excitatory corticostriatal terminals to the striatal MSNs (Pathway 1), with potential minor expression in inhibitory projections to striatal MSNs (Pathway 3) as well as excitatory projections to striatal PV interneurons (Pathway 2). Thus, these three M2-DLS/DMS projection pathways could be directly affected by optogenetic inhibition.

Several mechanisms could account for this unexpected synaptic response. First, feed-forward disinhibition of excitatory afferents: optogenetic inhibition of M2 terminals projecting to striatal PV interneurons (pathway 2) may reduce recruitment of these local inhibitory interneurons, thereby weakening their presynaptic suppression of M2 (pathway 5) or non-M2 (e.g., thalamostriatal; pathway 7) excitatory inputs, resulting in enhanced glutamate release from both sources. Second, anion dynamics specific to eNpHR in pathways 1 and 2: sustained Cl⁻ loading during prolonged light epochs (e.g., 1-min illumination) might shift the chloride reversal potential (E_Cl_), the voltage at which chloride currents through GABA_A_ receptors reverse, such that GABAergic inhibition is weakened or even inverted. This could reduce GABA_A_ receptor-mediated inhibition onto MSNs (pathway 5) and presynaptic sites (pathways 3, 6, 7), or trigger rebound excitation within the local circuit. These effects may in turn promote spillover-driven glutamate release from adjacent non-M2 afferents (pathway 4). Third, homeostatic recruitment of parallel non-M2 excitatory inputs (e.g., thalamostriatal; pathway 4) could compensate for the inhibition of M2-MSN excitatory inputs (pathway 1), further enhancing non-M2 presynaptic glutamate drive. In addition, mechanisms not illustrated in Fig. 3B (e.g., such as modulation by striatal cholinergic interneurons) [31, 32] may further contribute in complex ways.

Parrish et al. have shown that prolonged, intense halorhodopsin activation can induce ionic redistribution, including secondary K^+^ flux, which may lead to depolarization [33]. Thus, prolonged illumination could potentially influence synaptic transmission beyond direct terminal hyperpolarization. However, our data demonstrated that the increase in sEPSC frequency in DLS and DMS MSNs was most pronounced within the first ~10 s of the initial 1-min light-on period and diminished during the remaining 50 s (Fig. S1), a pattern inconsistent with progressive ionic accumulation during sustained illumination. Interestingly, after the first light-on epoch, sEPSC frequency returned to baseline during light-off, whereas stabilization at elevated levels emerged only after repeated light cycles (Fig. 4H, Q). This temporal profile suggests that if secondary K^+^ redistribution contributes to the observed synaptic effects of light inhibition, it is unlikely to be an acute consequence of a single illumination period, but may instead reflect a cumulative or adaptive process following repeated prolonged light-on sessions.

It is also worth noting that homeostatic mechanisms coupling intrinsic excitability of M2 cortical pyramidal neurons with presynaptic transmitter release (pathway 1) may explain why increased excitability of M2 cortical pyramidal neurons does not always guarantee enhanced glutamate release at their corticostriatal terminals onto MSNs. Such regulation could provide a safeguard against runaway excitation in corticostriatal circuits. Taken together, the increased sEPSC frequency may reflect enhanced recruitment of non-M2 excitatory inputs and/or altered inhibitory gating, rather than a simple increase in M2-derived glutamate release. In addition, structural postsynaptic changes, such as increased synapse number, altered spine density, or unsilencing of previously silent synapses, could also contribute to elevated sEPSC frequency and cannot be distinguished in the present recordings.

Different from most optogenetic studies that record effects within a specific circuit, our design combines circuit-specific presynaptic inhibition with non-specific postsynaptic recordings. Specifically, optogenetic inhibition targeted eNpHR-expressing M2 terminals, ensuring pathway specificity, while sEPSCs and sIPSCs recorded from MSNs reflected the integrated excitatory and inhibitory input from both M2 and non-M2 sources. Rather than a weakness, this approach is a strength, as it captures how M2 inputs interact with broader striatal networks (Fig. 3B). This strategy provides a more comprehensive understanding of how pathway-specific M2 changes influence relapse vulnerability. Future studies using chemogenetic inhibition may offer an alternative approach to further explore the functional organization of M2-striatal projections.

### Pathway-specific alterations in inhibitory transmission

Inhibitory responses diverged markedly between the DLS and DMS. In the DLS, repeated optogenetic inhibition increased sIPSC frequency across multiple epochs, suggesting persistent recruitment of local GABAergic inhibition (pathway 5) downstream of M2 input. In the DMS, however, inhibitory enhancement was transient: an initial increase in sIPSC frequency and amplitude was rapidly lost, with amplitude shifting toward attenuation. This fragility of inhibitory control (pathways 3, 5-7) may underline why silencing M2-DMS projections paradoxically enhances, rather than suppresses, cocaine seeking.

### Temporal divergence of inhibitory regulation in DLS versus DMS

Despite similar enhancement of excitatory transmission in both regions, inhibitory dynamics diverged markedly between the DLS and DMS. In the DLS, sIPSC frequency increased during the initial light-on period and remained elevated across subsequent inhibition cycles, indicating persistent recruitment of inhibitory control. In contrast, in the DMS, inhibitory enhancement was transient: sIPSC frequency and amplitude increased during the first light-on epoch but rapidly declined thereafter.

This temporal divergence in inhibitory persistence is likely to differentially shape MSN output. In the DLS, sustained inhibitory engagement may constrain MSN firing despite enhanced excitatory drive, consistent with reduced cocaine seeking following M2–DLS inhibition. In the DMS, rapid loss of inhibitory control would permit sustained excitation to dominate, potentially facilitating cocaine seeking when M2–DMS projections are inhibited. Thus, behavioral divergence appears to be determined not only by excitatory drive alone, but also by the persistence vs. transience of inhibitory regulation.

### Implications for relapse vulnerability

Together, these findings suggest that prolonged withdrawal reorganizes corticostriatal circuits in a pathway-specific manner. Hyperexcitable M2 neurons may amplify glutamatergic drive to both DLS and DMS (pathway 1), but the downstream balance of excitation and inhibition diverges. In the DLS, M2 inputs could recruit stable inhibitory control (pathways 3, 5-7) alongside excitatory enhancement, reinforcing habitual or compulsive drug seeking. In the DMS, inhibitory stabilization is rapidly lost, allowing for excess excitation (pathway 4) to dominate. This imbalance likely contributes to relapse vulnerability by disrupting the normal division of labor between striatal subregions and creating conditions in which M2-DMS suppression paradoxically promotes cocaine seeking.

## Conclusion

Our study highlights the complexity of MSN synaptic inputs and the need to consider both excitatory and inhibitory transmission when dissecting corticostriatal adaptations to cocaine. Importantly, optogenetic inhibition of eNpHR-expressing projections may not yield purely inhibitory outcomes, as their effects can be shaped by feed-forward and feedback loops that integrate excitatory and inhibitory circuits across local and long-range networks. Together, these findings emphasize that corticostriatal circuits are not uniform conduits of cortical output, but specialized pathways whose differential adaptations critically shape relapse risk.

## Supplementary information

Supplementary Information (clean version)

## Data Availability

The data that support the findings of this study are available from the corresponding author upon reasonable request.

## References

[1] Grimm JW, Hope BT, Wise RA, Shaham Y. Neuroadaptation. Incubation of cocaine craving after withdrawal. Nature. 2001;412:141–2. 11449260 10.1038/35084134 PMC2889613

[2] Yalachkov Y, Kaiser J, Naumer MJ. Sensory and motor aspects of addiction. Behav Brain Res. 2010;207:215–22. 19751771 10.1016/j.bbr.2009.09.015

[3] Wang L, Gao M, Wang Q, Sun L, Younus M, Ma S, et al. Cocaine induces locomotor sensitization through a dopamine-dependent VTA-mPFC-FrA cortico-cortical pathway in male mice. Nat Commun. 2023;14:1568. 36944634 10.1038/s41467-023-37045-3 PMC10030897

[4] Brown TE, Lee BR, Mu P, Ferguson D, Dietz D, Ohnishi YN, et al. A silent synapse-based mechanism for cocaine-induced locomotor sensitization. J Neurosci. 2011;31:8163–74. 21632938 10.1523/JNEUROSCI.0016-11.2011 PMC3286116

[5] Epstein DH, Preston KL, Stewart J, Shaham Y. Toward a model of drug relapse: an assessment of the validity of the reinstatement procedure. Psychopharmacol. 2006;189:1–16. 10.1007/s00213-006-0529-6 PMC1618790 17019567

[6] Corbit VL, Manning EE, Gittis AH, Ahmari SE. Strengthened inputs from secondary motor cortex to striatum in a mouse model of compulsive behavior. J Neurosci. 2019;39:2965–75. 30737313 10.1523/JNEUROSCI.1728-18.2018 PMC6462450

[7] Chen Y, Fu H, Korada A, Lange MA, Rayanki C, Lu T, et al. Decoding secondary motor cortex neuronal activity during cocaine self-administration: insights from longitudinal in vivo calcium imaging. Biol Psychiatry Glob Open Sci 2025. 10.1101/2024.05.20.594996 PMC12284276 40703665

[8] Korada A, Chen Y, Bi Z, Fu H, Lange MA, Montgomery JMF, et al. Temporal and spatial patterns of secondary motor cortex calcium activity in cocaine self-administration: A study using miniScope imaging and machine learning. Neuropharmacology. 2025;278:110574. 40578677 10.1016/j.neuropharm.2025.110574

[9] Huang D, Ma YY. Increased excitability of layer 2 cortical pyramidal neurons in the supplementary motor cortex underlies high cocaine-seeking behaviors. Biol Psychiatry. 2023;94:875–87. 37330163 10.1016/j.biopsych.2023.06.002 PMC10721734

[10] Yin HH, Knowlton BJ. The role of the basal ganglia in habit formation. Nat Rev Neurosci. 2006;7:464–76. 16715055 10.1038/nrn1919

[11] Roselli V, Guo C, Huang D, Wen D, Zona D, Liang T, et al. Prenatal alcohol exposure reduces posterior dorsomedial striatum excitability and motivation in a sex- and age-dependent fashion. Neuropharmacology. 2020;180:108310. 32950559 10.1016/j.neuropharm.2020.108310 PMC7773417

[12] Murray JE, Belin D, Everitt BJ. Double dissociation of the dorsomedial and dorsolateral striatal control over the acquisition and performance of cocaine seeking. Neuropsychopharmacology. 2012;37:2456–66. 22739470 10.1038/npp.2012.104 PMC3442340

[13] Belin D, Everitt BJ. Cocaine seeking habits depend upon dopamine-dependent serial connectivity linking the ventral with the dorsal striatum. Neuron. 2008;57:432–41. 18255035 10.1016/j.neuron.2007.12.019

[14] Bender BN, Stringfield SJ, Torregrossa MM. Changes in dorsomedial striatum activity during expression of goal-directed vs. habit-like cue-induced cocaine seeking. Addict Neurosci. 2024;11:100149. 10.1016/j.addicn.2024.100149 PMC11218864 38957402

[15] Kalivas PW. The glutamate homeostasis hypothesis of addiction. Nat Rev Neurosci. 2009;10:561–72. 19571793 10.1038/nrn2515

[16] Ma YY, Lee BR, Wang X, Guo C, Liu L, Cui R, et al. Bidirectional modulation of incubation of cocaine craving by silent synapse-based remodeling of prefrontal cortex to accumbens projections. Neuron. 2014;83:1453–67. 25199705 10.1016/j.neuron.2014.08.023 PMC4295617

[17] Conrad KL, Tseng KY, Uejima JL, Reimers JM, Heng LJ, Shaham Y, et al. Formation of accumbens GluR2-lacking AMPA receptors mediates incubation of cocaine craving. Nature. 2008;454:118–21. 18500330 10.1038/nature06995 PMC2574981

[18] Lu L, Grimm JW, Hope BT, Shaham Y. Incubation of cocaine craving after withdrawal: a review of preclinical data. Neuropharmacology. 2004;47:214–26. 15464139 10.1016/j.neuropharm.2004.06.027

[19] Huang D, Ma YY. Downregulation of small conductance potassium channels in cortical pyramidal neurons of the supplementary motor cortex underlies high cocaine seeking behaviors. Biol Psychiatry. 2025. 10.1016/j.biopsych.2025.10.037. 41232897

[20] Dong Y, Nasif FJ, Tsui JJ, Ju WY, Cooper DC, Hu XT, et al. Cocaine-induced plasticity of intrinsic membrane properties in prefrontal cortex pyramidal neurons: adaptations in potassium currents. J Neurosci. 2005;25:936–40. 15673674 10.1523/JNEUROSCI.4715-04.2005 PMC6725625

[21] Tepper JM, Koos T, Ibanez-Sandoval O, Tecuapetla F, Faust TW, Assous M. Heterogeneity and diversity of striatal GABAergic interneurons: update 2018. Front Neuroanat. 2018;12:91. 30467465 10.3389/fnana.2018.00091 PMC6235948

[22] Urrutia-Pinones J, Morales-Moraga C, Sanguinetti-González N, Escobar AP, Chiu CQ. Long-Range GABAergic projections of cortical origin in brain function. Front Syst Neurosci. 2022;16:841869. 35392440 10.3389/fnsys.2022.841869 PMC8981584

[23] Melzer S, Gil M, Koser DE, Michael M, Huang KW, Monyer H. Distinct corticostriatal GABAergic neurons modulate striatal output neurons and motor activity. Cell Rep. 2017;19:1045–55. 28467898 10.1016/j.celrep.2017.04.024 PMC5437725

[24] Rock C, Zurita H, Wilson C, Apicella AJ. An inhibitory corticostriatal pathway. Elife. 2016;5:e15890. 10.7554/eLife.15890 PMC4905740 27159237

[25] Klug JR, Engelhardt MD, Cadman CN, Li H, Smith JB, Ayala S, et al. Differential inputs to striatal cholinergic and parvalbumin interneurons imply functional distinctions. Elife. 2018;7:e35657. 10.7554/eLife.35657 PMC5929909 29714166

[26] Namkung H, Kim SH, Sawa A. The Insula: an underestimated brain area in clinical neuroscience, psychiatry, and neurology. Trends Neurosci. 2017;40:200–07. 28314446 10.1016/j.tins.2017.02.002 PMC5538352

[27] Martel AC, Galvan A. Connectivity of the corticostriatal and thalamostriatal systems in normal and Parkinsonian states: an update. Neurobiol Dis. 2022;174:105878. 36183947 10.1016/j.nbd.2022.105878 PMC9976706

[28] Hunnicutt BJ, Jongbloets BC, Birdsong WT, Gertz KJ, Zhong H, Mao T. A comprehensive excitatory input map of the striatum reveals novel functional organization. Elife. 2016;5:e19103. 10.7554/eLife.19103 PMC5207773 27892854

[29] Dittgen T, Nimmerjahn A, Komai S, Licznerski P, Waters J, Margrie TW, et al. Lentivirus-based genetic manipulations of cortical neurons and their optical and electrophysiological monitoring in vivo. Proc Natl Acad Sci USA. 2004;101:18206–11. 15608064 10.1073/pnas.0407976101 PMC539748

[30] Veres JM, Andrasi T, Nagy-Pal P, Hajos N. CaMKIIalpha promoter-controlled circuit manipulations target both pyramidal cells and inhibitory interneurons in cortical networks. eNeuro. 2023;10: ENEURO.0070-23. 10.1523/ENEURO.0070-23.2023 PMC10088982 36963833

[31] Doig NM, Magill PJ, Apicella P, Bolam JP, Sharott A. Cortical and thalamic excitation mediate the multiphasic responses of striatal cholinergic interneurons to motivationally salient stimuli. J Neurosci. 2014;34:3101–17. 24553950 10.1523/JNEUROSCI.4627-13.2014 PMC3931511

[32] Fritz M, Rosa PB, Wilhelms D, Jaarola M, Ruud J, Engblom D, et al. Nicotinic alpha7 receptors on cholinergic neurons in the striatum mediate cocaine reinforcement, but not food reward. Front Mol Neurosci. 2024;17:1418686. 39906479 10.3389/fnmol.2024.1418686 PMC11790553

[33] Parrish RR, MacKenzie-Gray Scott C, Jackson-Taylor T, Grundmann A, McLeod F, Codadu NK, et al. Indirect effects of halorhodopsin activation: potassium redistribution, nonspecific inhibition, and spreading depolarization. J Neurosci. 2023;43:685–92. 36639898 10.1523/JNEUROSCI.1141-22.2022 PMC9899079

