# Supplementary material for: Contrasting roles of secondary motor cortex projections to the dorsolateral and dorsomedial striatum in incubation of cocaine-seeking
Source: Neuropsychopharmacology. 2026 Mar 25;51(11):2206–16. doi: 10.1038/s41386-026-02385-3 (PMC13597483; doi:10.1038/s41386-026-02385-3)
Supplement: Supplementary file 1 [file 41386_2026_2385_MOESM1_ESM.docx]

**SUPPLEMENTARY INFORMATION**

**Contrasting Roles of Secondary Motor Cortex Projections to the Dorsolateral and Dorsomedial Striatum in Incubation of Cocaine-Seeking**

Víctor Gómez-Pineda (PH.D.)^1^, Donald Huang (PH.D.)^1^, Yingying Chen (PH.D.)^1^, Changyong Guo (M.D., PH.D.)^1^, Yao-Ying Ma (M.D., PH.D.)^1,2*^

^1^, Department of Biochemistry, Molecular Biology and Pharmacology, Indiana University School of Medicine, Indianapolis, IN 46202, USA.

^2^, Stark Neurosciences Research Institute, Indiana University School of Medicine, Indianapolis, IN 46202, USA.

***Corresponding author**: Dr. Yao-Ying Ma

Department of Biochemistry, Molecular Biology, and Pharmacology, Indiana University School of Medicine, Indianapolis, IN 46202, USA.

**MATERIALS AND METHODS**

**Experimental Subjects**

All experiments were conducted in accordance with the United States Public Health Service Guide for Care and Use of Laboratory Animals and were approved by the Institutional Animal Care and Use Committee at Indiana University School of Medicine. Experimental procedures were performed on inbred male Sprague-Dawley rats using breeders originating from Envigo (USA) or purchased from Envigo one week prior to experimentation. All rats were maintained on a 12-hour light/dark cycle (lights on at 7:00 AM, off at 7:00 PM), housed in clear Plexiglas bins, and given ad libitum access to food and water except during operant training. The day of birth was designated as postnatal day (P) 0. Rat pups developed undisturbed until weaned on P21-23. After weaning, they were pair-housed until catheter implantation at the age of 5 weeks old, after which they were single-housed. A total of 140 rats were used in this study; 22 were excluded due to catheter patency failure (n = 9), overnight session failure (n = 4; fewer than 80 intravenous cocaine infusions even after the third overnight session), poor or off-site AAV expression (n = 5), or off-target implantation / damage of optic fibers (n = 4).

**Surgery procedures**

*Catheter implantation*

As reported in previous works from our lab [1-3], a silastic catheter was inserted into the right jugular vein, and the distal end was led subcutaneously to the back between the scapulae. Catheters were constructed from silastic tubing (~8 cm; inner diameter 0.020 in, outer diameter 0.037 in) connected to a One Channel Rat Button (Instech Labs). Rats were allowed to recover for ~7 days. During recovery, the catheter was flushed daily with 1 ml/kg body weight of heparin (10 U/ml) and gentamicin antibiotic (5 mg/ml) in sterile saline to help protect against infection and catheter occlusion.

*Microinjection of AAV*

AAV injections were performed immediately after catheter implantation within the same surgical session, with stereotaxic guidance provided by a Kopf apparatus. A 28-gauge injection needle was used to bilaterally inject 1 µl/site (0.2 µl/min) of the AAV5-CaMKIIα-eNpHR3.0-mCherry or AAV5-CaMKIIα-mCherry solution (UNC Vector Core) *via* a Hamilton syringe into the M2 region (in mm: AP, +2.4; ML, ±1.3; DV as the distance from the skull surface, -1.5), using the Pump 11 Elite Syringe Pumps (Harvard Apparatus). Injection needles were left in place for 5 min following injection.

*Implantation of optic fibers*

As reported previously [4,5], seven days before the cocaine seeking test on withdrawal day 38, rats assigned to *in vivo* optogenetic inhibition experiments were anesthetized with vaporized isoflurane (2-3%) and positioned in a stereotaxic apparatus (Kopf). Bilateral optic fibers were implanted into the DLS (AP: +0.5 mm, ML: ±4.0 mm, DV: -4.5 mm) or the DMS (AP: +0.5 mm, ML: ±2.1 mm, DV: -3.7 mm). Optic fiber cannulas (Ø200 μm; Doric Lenses) were secured to the skull using sterile stainless-steel screws (Plastics One) and dental cement (Ortho-Jet). Following surgery, a plastic cap was placed to protect the fiber tip. For habituation, during the three days prior to *in vivo* optogenetic inhibition, the cap was removed daily, and the optic fiber tip was connected to a patch cord.

**Intravenous self-administration of cocaine**

*Self-administration apparatus:*

Behavioral experiments were conducted in operant-conditioning chambers enclosed within sound-attenuating cabinets (Med Associates). The chambers were equipped with a house light, two levers (randomly assigned as active *vs*. inactive), two conditioned stimulus lights (i.e., cue lights) 9 cm above each lever, and a food dispenser. Water and food were absent during training and testing sessions.

*Intravenous cocaine self-administration training:*

Intravenous self-administration training began with 1-3 overnight sessions from 7 PM to 7 AM on the following day. The daily training session, i.e., 1 hour per day for 5 days, started on the day after. The same training protocol was used in overnight and daily sessions. Rats were placed in the self-administration chamber on a fixed ratio (FR) 1 reinforcement schedule with the house light on. Active lever presses resulted in a cocaine infusion (0.75 mg/kg over 2-4 sec) and illumination of a cue light above the active lever for 20 sec with the house light off. In contrast, the inactive lever presses led to no outcome but were also recorded. Rats that received at least 80 cocaine rewards in the overnight session were allowed to move to daily self-administration of cocaine ~24 hr after the overnight training.

*Cocaine seeking test:*

Rats previously assigned for behavioral examination were tested on either withdrawal day 1 or withdrawal day 45 for their cocaine seeking behaviors (**Figs. 1E, 2**). The cocaine-seeking test lasted 1 hr, during which rats were re-exposed to the operant chamber with the exact same conditions as during cocaine self-administration training except no cocaine IV delivery was programmed after active lever presses. The active lever press during 1 hr seeking test was analyzed as an index of cocaine seeking level.

*Effects of in vivo Optogenetic inhibition* *on cocaine seeking:*

The external end of the pre-implanted optic fiber cannula was connected to a Ce:YAG + LED/LD Fiber Light Source (Doric Lenses) *via* a patch cord prior to the 1-hr cocaine-seeking test on withdrawal day 45. During the test, rats received bilateral optogenetic inhibition (590 nm, 5 mW optical output per fiber tip) delivered in a 1-min on/1-min off pattern, programmed using Doric Neuroscience Studio. Sham control rats were connected to the light source and received inhibition under identical parameters, except that the power was attenuated to 1% output (0.05 mW per fiber tip).

**Open field test**

Black Plexiglas open field testing chamber (Maze Engineers) was 60 cm for both side-widths x 50 cm in height with a floor illumination at ~60-80 lux [6]. Rats were placed in the peripheral area facing one of the randomly assigned chamber’s sidewalls and then allowed to freely explore the chamber. Total testing time was 5 min. Behavioral videos were processed by EthoVision XT14 Software (Noldus). The open field test was done two days after cocaine seeking test.

**Verification of the AAV expression and optic fiber location**

Following the open-field test, brains were fixed by transcardial perfusion with 4% paraformaldehyde (PFA), and coronal slices (50 µm) containing either the M2 or DLS/DMS were prepared. After rinsing in PBS, slices were mounted with ProLong™ Gold Antifade Mountant with DAPI (Invitrogen, Cat# P36931). Confocal imaging was performed using a Zeiss LSM 800 microscope to verify AAV expression and confirm the placement of optic fibers as described previously [2].

**Brain slice and whole-cell patch clamp recordings**

*Slice preparation:*

Brain slices were prepared using standard procedures for whole-cell patch clamp recordings as detailed in our previous publications [7-9]. Before sacrifice, rats were anesthetized with isoflurane and subsequently transcardially perfused with 4°C cutting solution (in mM: 135 *N*-methyl-D-glucamine, 1 KCl, 1.2 KH_2_PO_4_, 0.5 CaCl_2_, 1.5 MgCl_2_, 20 choline-HCO_3_, 11 glucose, pH adjusted to 7.4 with HCl, and saturated with 95% O_2_ /5% CO_2_). The rat was decapitated, and then the brain was removed and glued to a block before slicing using a Leica VT1200s vibratome in 4°C cutting solution. Coronal slices of 250-µm thickness were cut such that the preparation contained the signature anatomical landmarks (e.g., the corpus callosum, the lateral ventricle, the striatum, and the anterior commissure) that delineate the brain areas of interest. After allowing at least 1 hr for recovery, slices were transferred from a holding chamber to a submerged recording chamber where it was continuously perfused with oxygenated ACSF, containing (in mM) 119 NaCl, 2.5 KCl, 2.5 CaCl_2_, 1.3 MgCl_2_, 1 NaH_2_PO_4_, 26.2 NaHCO_3_, and 11 glucose and maintained at 30 ± 1°C. Picrotoxin (100 µM; Sigma-Aldrich) was included in ACSF bath to block GABA_A_ receptors during sEPSC recording, and APV (50 µM; Sigma-Aldrich) and CNQX (10 µM; Sigma-Aldrich) were added to block NMDA and AMPA receptors during sIPSC recordings. Yellow light (λ = 590 nm; pE-300 Ultra LED, CoolLED) was delivered at 75% of the maximum output through a 40× water-immersion objective for the time window specified in each experiment.

*Intrinsic excitability recordings:*

Patch electrodes contained (in mM): 108 KMeSO_3_, 20 KCl, 0.4 K-EGTA, 10 HEPES, 2.5 Mg-ATP, 0.25 Na-GTP, 7.5 phosphocreatine (Na2), 1 L-glutathione, 2 MgCl_2_, pH 7.3. Layer 2 cortical projection neurons in M2 were first patched in voltage-clamp mode and then switched to current-clamp mode. The membrane potential was adjusted to −70 mV by applying a depolarizing holding current (50-200 pA) throughout the current-clamp recordings. To evaluate neuronal excitability, a series of 300-ms current injection steps ranging from +50 to +400 pA in 50 pA increments was applied, as previously described [10,11]. The effects of optogenetic inhibition on intrinsic excitability of eNpHR-expressing M2 cortical pyramidal neurons were evaluated using yellow light (λ = 590 nm, pE-300 Ultra LED, CoolLED) delivered at 15% of the maximum output, applied 1 s before the onset of each current-clamp sweep and terminating concurrently with sweep completion.

*sEPSC and sIPSC recordings and effects of optogenetics:*

Patch electrodes (3-5 MΩ) were filled with an internal solution containing (in mM): 125 CsCH_3_O_3_S, 15 CsCl, 5 TEA-Cl, 0.4 EGTA, 20 HEPES, 2.5 Mg-ATP, 0.25 Na-GTP, 1 QX-314, pH 7.3. Cells were patched in voltage-clamp mode and held at −70 mV. Membrane capacitance (Cm), input resistance (Rm), and time constant (Tau) were measured by applying a 5-mV depolarizing pulse and analyzed using the membrane test function in Clampex 11 to confirm passive membrane properties. sEPSCs and sIPSCs were recorded in gap-free voltage-clamp mode with a holding voltage at -70 mV and +10 mV, respectively. Adopting the protocol previously described [12], the effects of optogenetic inhibition on sEPSCs and sIPSCs were evaluated by exciting eNpHR-expressing M2 terminals in the DLS or DMS. Yellow light (∼590 nm, pE-300 Ultra LED, CoolLED) was applied for 1 min following at least 1 min of baseline recording. Light inhibition was repeated for up to three rounds, each separated by a 1-min light-off interval. sEPSCs and sIPSCs were analyzed offline with MiniAnalysis (Synapto Soft). Thresholds for detection were set at 6 pA for sEPSCs and 10 pA for sIPSCs.

**Statistical Analysis:** Data were collected from at least 8 rats per group for behavioral evaluations, and from at least 6 rats (1-3 neurons per rat) per group for whole-cell patch-clamp recordings. Results are presented as mean ± SEM and/or as individual values from each neuron or each rat. Using GraphPad Prism 10.6, statistical significance was assessed by Student t test (**Fig. 2J,K**), and one-way ANOVA (**Fig. 1U**) or two-way ANOVA (**Figs. 1B-E,I,O,P,T;2C-E,G-I; 3C,D,F,G,I,J,L,M; 4B,C,E,F,H,I,K,L; 5B,C,E,F; S2A-D**), followed by Bonferroni post-hoc tests. Statistical significance was achieved if p < 0.05.

**SUPPLEMENTARY FIGURES**

**Figure S1**. **Effects of optogenetic inhibition of M2-striatal projections with varying durations on MSN sEPSCs, analyzed in 10-second binned time windows on withdrawal day 45 in cocaine-treated rats**

**A, B**, Summary data showing optogenetic inhibition at 1 min, but not 1 sec or 100 ms, enhanced the sEPSC frequency (**A**, inhibition duration × inhibition timing interaction F_22,297_=3.1, p<0.01; inhibition duration F_2,27_=2.1, p=0.14; inhibition timing F_6,159_=5.1, p<0.01), and similar amplitude (**B**, inhibition duration × inhibition timing interaction F_22,297_=1.1, p=0.34; inhibition duration F_2,27_=1.0, p=0.38; inhibition timing F_5,141_=1.0, p=0.41) in DLS MSNs on withdrawal day 45 in cocaine rats.

**C,D**, Summary data showing optogenetic inhibition at 1 min, but not 1 sec or 100 ms, enhanced the sEPSC frequency (**C**, inhibition duration × inhibition timing interaction F_22,242_=1.6, p=0.046; inhibition duration F_2,22_=5.2, p=0.01; inhibition timing F_4,96_=3.2, p=0.01), and similar amplitude (**D**, inhibition duration × inhibition timing interaction F_22,253_=0.7, p=0.82; inhibition duration F_2,23_=3.1, p=0.07; inhibition timing F_5,104_=0.6, p=0.71) in DMS MSNs on withdrawal day 45 in cocaine rats.

Data were collected from m neurons in n rats as expressed in m/n in figure legends for each group, and analyzed by two-way ANOVA with repeated measures on inhibition timing, followed by Bonferroni’s post hoc test. *, p<0.05, **, p<0.01, between-neuron comparisons at the same inhibition time bin relative to the 100 ms in purple and relative to 1 sec in green. #, p<0.05, ##, p<0.01, within-neuron comparisons relative to any time bin before light inhibition onset.

**REFERENCES**

1	Guo C, Wen D, Zhang Y, Mustaklem R, Mustaklem B, Zhou M, et al. Amyloid-beta oligomers in the nucleus accumbens decrease motivation via insertion of calcium-permeable AMPA receptors. Mol Psychiatry. 2022;27(4):2146-57.

2	Huang D, Ma YY. Increased Excitability of Layer 2 Cortical Pyramidal Neurons in the Supplementary Motor Cortex Underlies High Cocaine-Seeking Behaviors. Biol Psychiatry. 2023;94(11):875-87.

3	Luo YX, Huang D, Guo C, Ma YY. Limited versus extended cocaine intravenous self-administration: Behavioral effects and electrophysiological changes in insular cortex. CNS Neurosci Ther. 2021;27(2):196-205.

4	Ma YY, Lee BR, Wang X, Guo C, Liu L, Cui R, et al. Bidirectional modulation of incubation of cocaine craving by silent synapse-based remodeling of prefrontal cortex to accumbens projections. Neuron. 2014;83(6):1453-67.

5	Ma YY, Wang X, Huang Y, Marie H, Nestler EJ, Schluter OM, et al. Re-silencing of silent synapses unmasks anti-relapse effects of environmental enrichment. Proceedings of the National Academy of Sciences of the United States of America. 2016;113(18):5089-94.

6	Shan L, Galaj E, Ma YY. Nucleus accumbens shell small conductance potassium channels underlie adolescent ethanol exposure-induced anxiety. Neuropsychopharmacology : official publication of the American College of Neuropsychopharmacology. 2019.

7	Galaj E, Guo C, Huang D, Ranaldi R, Ma YY. Contrasting effects of adolescent and early-adult ethanol exposure on prelimbic cortical pyramidal neurons. Drug Alcohol Depend. 2020;216:108309.

8	Luo YX, Galaj E, Ma YY. Differential alterations of insular cortex excitability after adolescent or adult chronic intermittent ethanol administration in male rats. J Neurosci Res. 2021;99(2):649-61.

9	Roselli V, Guo C, Huang D, Wen D, Zona D, Liang T, et al. Prenatal alcohol exposure reduces posterior dorsomedial striatum excitability and motivation in a sex- and age-dependent fashion. Neuropharmacology. 2020;180:108310.

10	Shan L, Galaj E, Ma YY. Nucleus accumbens shell small conductance potassium channels underlie adolescent ethanol exposure-induced anxiety. Neuropsychopharmacology : official publication of the American College of Neuropsychopharmacology. 2019;44(11):1886-95.

11	Ma YY, Cepeda C, Chatta P, Franklin L, Evans CJ, Levine MS. Regional and cell-type-specific effects of DAMGO on striatal D1 and D2 dopamine receptor-expressing medium-sized spiny neurons. ASN neuro. 2012;4(2).

12	Holley SM, Galvan L, Kamdjou T, Dong A, Levine MS, Cepeda C. Major Contribution of Somatostatin-Expressing Interneurons and Cannabinoid Receptors to Increased GABA Synaptic Activity in the Striatum of Huntington's Disease Mice. Front Synaptic Neurosci. 2019;11:14.
